# Automated UMLS-Based Comparison of Medical Forms

**DOI:** 10.1371/journal.pone.0067883

**Published:** 2013-07-04

**Authors:** Martin Dugas, Fleur Fritz, Rainer Krumm, Bernhard Breil

**Affiliations:** Institute of Medical Informatics, University of Münster, Münster, Germany; Rensselaer Polytechnic Institute, United States of America

## Abstract

Medical forms are very heterogeneous: on a European scale there are thousands of data items in several hundred different systems. To enable data exchange for clinical care and research purposes there is a need to develop interoperable documentation systems with harmonized forms for data capture. A prerequisite in this harmonization process is comparison of forms. So far – to our knowledge – an automated method for comparison of medical forms is not available. A form contains a list of data items with corresponding medical concepts. An automatic comparison needs data types, item names and especially item with these unique concept codes from medical terminologies. The scope of the proposed method is a comparison of these items by comparing their concept codes (coded in UMLS). Each data item is represented by item name, concept code and value domain. Two items are called identical, if item name, concept code and value domain are the same. Two items are called matching, if only concept code and value domain are the same. Two items are called similar, if their concept codes are the same, but the value domains are different. Based on these definitions an open-source implementation for automated comparison of medical forms in ODM format with UMLS-based semantic annotations was developed. It is available as package *compareODM* from http://cran.r-project.org. To evaluate this method, it was applied to a set of 7 real medical forms with 285 data items from a large public ODM repository with forms for different medical purposes (research, quality management, routine care). Comparison results were visualized with grid images and dendrograms. Automated comparison of semantically annotated medical forms is feasible. Dendrograms allow a view on clustered similar forms. The approach is scalable for a large set of real medical forms.

## Introduction

Medical documentation can be quite complex: For instance, a typical electronic health record (EHR) system can contain several hundred different forms [1]. Each form contains a list of data items, therefore a full-scale EHR can consist of ∼100.000 data items with an annual increase up to 6 % [2], [3]. In addition to this routine documentation there are several forms for research purpose in use, e.g. in electronic data capture (EDC) systems for clinical studies. In clinical trials, these case report forms (CRFs) can consist of hundreds of items per trial [4]. To make it even more complicated, all these different forms can change over time, for instance because of protocol amendments in clinical trials. Both from a data management and a data analysis perspective it is relevant to know which items were changed between different versions of these forms. Currently this comparison can only be performed manually.

There are several typical use cases where comparison of medical forms is beneficial:

- Design of new or update of existing clinical documentation forms
- Design/amendment of clinical trial documentation forms
- Identification of duplicated items to avoid redundant documentation
- Identification of items with potential for secondary use.

The scope of the proposed method is a comparison of items, annotated with medical concepts, between two given medical forms. Other aspects of forms, such as layout information (font size, color, number of pages, format, position of items etc.), are disregarded. The rationale for this approach is semantic interoperability, i.e. what data collected by these forms could potentially be exchanged or pooled. Typically, these forms evolve over time: Data items are added or removed, data types and/or value lists for these items are updated.

Currently for each hospital and each clinical trial medical forms are developed and maintained on an individual basis. Vendors of EHRs typically provide a set of standard medical forms (for example medical history, discharge letter); however, most of these forms are further customized in each hospital. In addition, there are many different EHR systems (∼900 EHR vendors in Europe [5]), therefore there are many different “standard” EHR form sets. In clinical trials, CRFs are managed within EDC systems. Given the large number of hospitals (more than 10.000 hospitals in Europe) and clinical trials (>100.000 registered trials [6]) there is a huge variety of medical forms. All forms contain items corresponding to medical concepts. Some studies show the potential of reuse by identifying the same concepts in different forms [7] which implies that the variety is probably more based on formal aspects and layout information than on different concepts. This diversity of medical forms severely hampers semantic interoperability between different information systems: it is very difficult to transfer structured patient data between different EHR systems or between EHR and EDC systems in a research context, because the two involved systems do not use the same metadata.

### Objectives

In order to enable data exchange, an analysis of forms is a basic activity while designing information systems and can provide insight to harmonize medical forms. Comparison of forms to identify identical, matching, similar or differing data items is an initial step of this analysis. Given the complexity of medical forms, manual comparison of two forms (either two versions of the same form or two different forms) can take quite a lot of work and is prone to error.

Therefore, we want to analyze, whether an automatic form comparison is feasible and whether it is scalable to a set of forms. In the following, we propose a method to enable automatic comparison of medical forms and evaluate its feasibility and scalability with a set of real medical forms.

## Methods

There are various electronic representations for medical forms available, in particular the Operational Data Model (ODM) from Clinical Data Interchange Standards Consortium (CDISC) [8] and the Clinical Document Architecture (CDA) from Health Level Seven (HL7) [9]. In addition, medical terminologies are needed for the specification of items on these forms.

### Item Definition

A single data item of a medical form can be described by its item name, data type, a concept and a value domain. ISO 11179 requires for each data item a unique identifier, a name according to the name principles, a definition according to the data definition rules and a classification [10]. Each data item represents a medical concept. In order to facilitate automatic form comparison it is important to use unique identifiers or preferably unique codes to specify these concepts. Such codes are provided by terminologies like the Unified Medical Language System (UMLS) [11] or Systematized Nomenclature of Medicine (SNOMED) [12]. For example, for the item named “patient diagnosis” one could use the UMLS code C011900 to refer to the concept “diagnosis”. The value domain can be characterized by a data type (for instance Boolean, Integer) or a list of items (codes) from a terminology. In our example an appropriate value domain would be “ICD10 version 2012 German modification” [13].

#### UMLS

The UMLS contains 2.7 million concepts from over 160 source vocabularies (as of June 2012). Via the NCI Metathesaurus [14] more than 1.4 million concept codes from 76 sources with mappings to UMLS concept codes are publicly available. Therefore, a very fine-granular semantic annotation of data items is feasible. In general, semantic annotation of data items can be performed as precoordination (one concept per item) or postcoordination (several concepts per item) [15].

### Form Definition

#### HL7 CDA

CDA is an HL7 standard [9] to describe structure and semantics of clinical documents. It is well established for EHR systems. For example, metadata of a CDA document contain information about author of the document and the clinical setting. The body specification of CDA Release 2 (R2) documents facilitates semantic interoperability [16] by defining three levels of computable codes to amend the narrative, human-readable text. On level 1 there is only unstructured text, on level 2 there are sections with codes, and on level 3 data items within these sections are coded. These codes are derived from HL7 reference information model (RIM) classes (for example observation or procedure). Several document types are based upon CDA, for example Continuity of Care Document (CCD) [17] and Clinical Research Document (CRD) [18].

#### CDISC ODM

CDISC is developing data standards for clinical research which are supported by regulatory agencies such as Food and Drug Administration (FDA) and European Medicines Agency (EMA). ODM is an XML-based CDISC-standard which is commonly used in clinical trials to represent – among other data structures – CRFs. ODM is more generic than clinical document architecture (CDA) regarding representation of forms, because it does not mandate certain section headings. ODM enables to define item groups, items and value lists for each item. Both, CDA and ODM are system independent and, importantly, can be semantically annotated [19].

### Compare Algorithm

#### Comparison of Two Forms

If forms are considered as lists of items, two forms can be compared by pairwise comparison of these items. In our reference implementation for each data item from form 1 the “best” fitting item within form 2 is identified and an aggregated report is generated. This report provides identical, matching, similar and differing items. It is programmed in R [20].

#### Comparison of Data Items

To compare data items an appropriate representation must be chosen. In the following, a single item shall be represented by item name, concept code and value domain.

Two items are called *identical*, if item name, concept code and value domain are the same. Note: Identical item names alone are not sufficient for item identity, for example an item named “size” might be size of the head in one context and size of the feet in another context. Therefore, concept codes and value domains are needed to check identity.

Two items are called *matching*, if item names are different, but concept codes and value domain are the same. For example, an item named “birth date” and an item “date of birth” would be called matching, if concept code and value domain are the same.

Two items are called *similar*, if their concept codes are the same, but value domains are different. For example, “serum creatinine” could have a value domain with measurement unit “mg/dl” and another value domain with “mmol/l”, respectively.

Two items, which are neither identical, nor matching, nor similar, are called *differing* items.

According to these definitions, data from identical or matching items could be pooled and analyzed jointly. Data from similar items need to be transformed before a joint analysis is possible.

### Evaluation

To assess the feasibility and scalability of the form comparison algorithm, it was applied to a set of forms from a repository of more than 3,500 forms with 102,000 items available in the CDISC ODM format. More than 80% of these items are coded with terminology codes [21]. The scope of the evaluation was a test with real medical forms that meet the following criteria and were manually selected out of the repository:

- At least 90% of the items were coded
- All forms are related to one medical disease, in this case prostate cancer (to expect at least some shared attributes)

All forms are compared pairwise and results are stored in three matrices (numbers of identical, matching and similar items) which are visualized. Cluster analyses were applied.

#### Visualization

In order to visualize the results of the group comparison, different visualization types are applied. Grid images create grids of colored rectangles with colors corresponding to the values in the respective matrix. Dendrogram are used for visualization of the compared forms in a tree diagram and show the relationships of similarity in a group of forms [22]. Dendrograms allow a hierarchical clustering and classification/regression of the results. In these tests the *image* and *hclust* functions from the R-repository are applied [20].

## Results

An open-source reference implementation of the proposed method to compare medical forms is available as package *compareODM* from http://cran.r-project.org. Input for this program are two forms in ODM format, output is a report regarding identical, matching, similar and differing items. The first part of the results describes results of the comparison for two forms. The second part presents how the method is applied to a set of forms.

### Comparison of Two Forms

**Figure 1:**
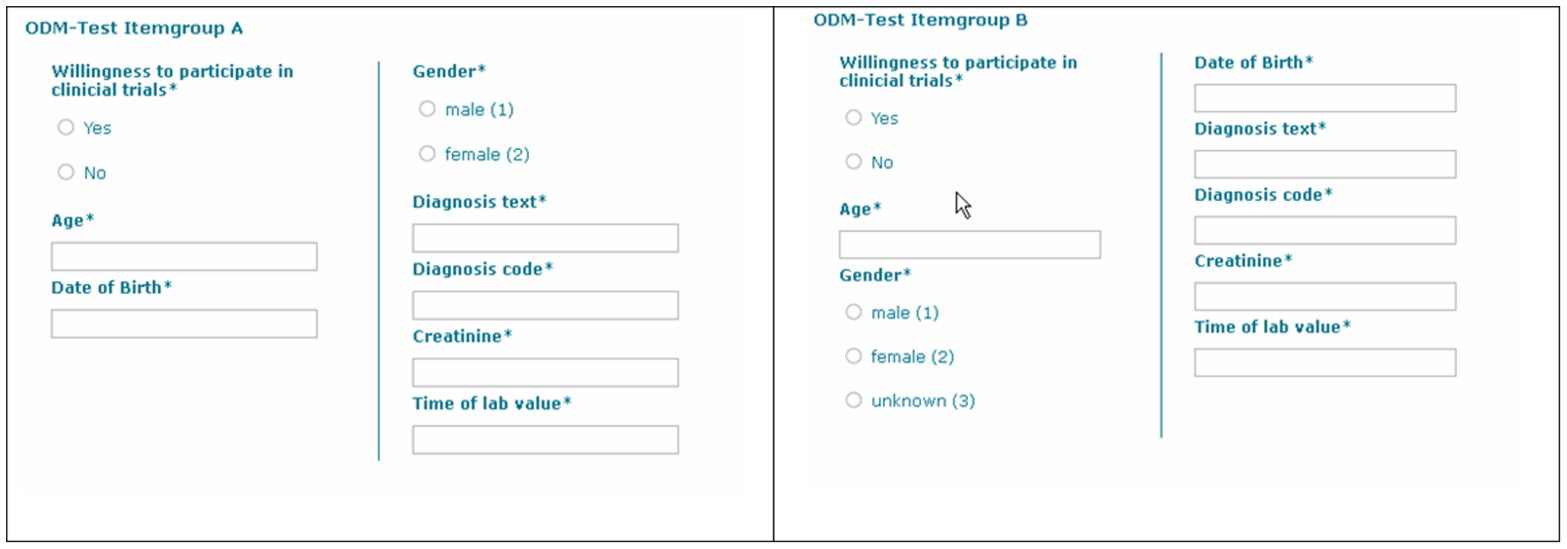
Overview of two simple forms with 8 items each. Different data types are available, for example categorical (“Willingness to participate in clinical trials”), integer (“Age”) and date (“Date of Birth”).

Figure 1 presents two simple examples of medical forms, which are available within our package *compareODM*. Each one consists of 8 data items. In this overview no details about concepts are provided. This figure presents the forms in a way a physician would use it for documentation.

**Figure 2:**
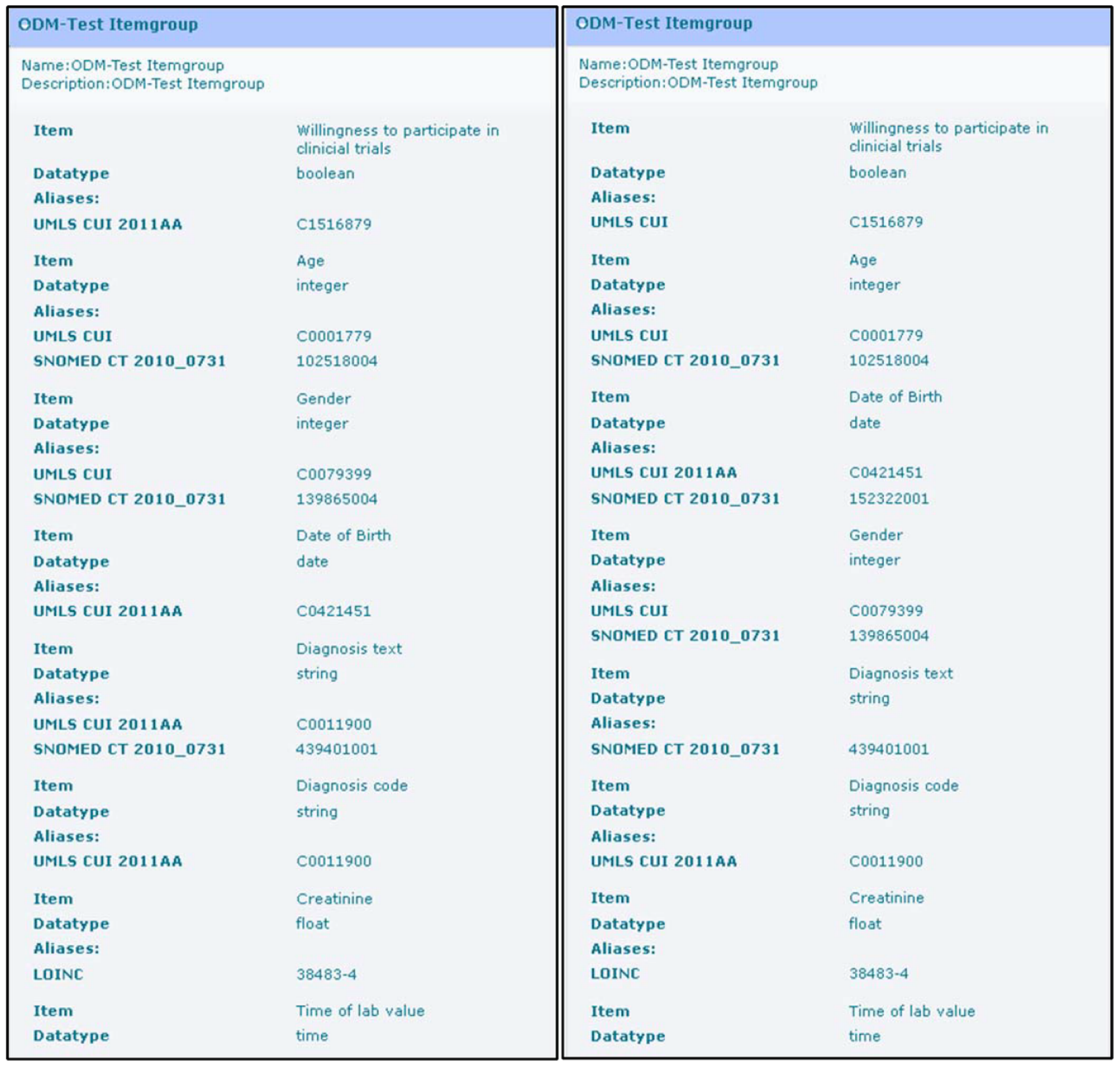
Detailed view of the forms from Fig. 1 with UMLS Concept Unique Ids (CUI). For instance, it can be seen that the first item of both forms has assigned the same CUI (C1516879). In addition, information about the value domain for each item is provided (for example: data type boolean).

For the described algorithm it is necessary to have ODM forms with coded items. Figure 2 presents a more detailed view of those forms, including UMLS concept IDs and information about the value domain (data type). ODM version 1.3 provides – among others – the following basic data types: Boolean, date, time, string and float. In addition, code lists can be defined in ODM for each item, for example regarding gender to define values for “male” and “female”.

**Figure 3:**
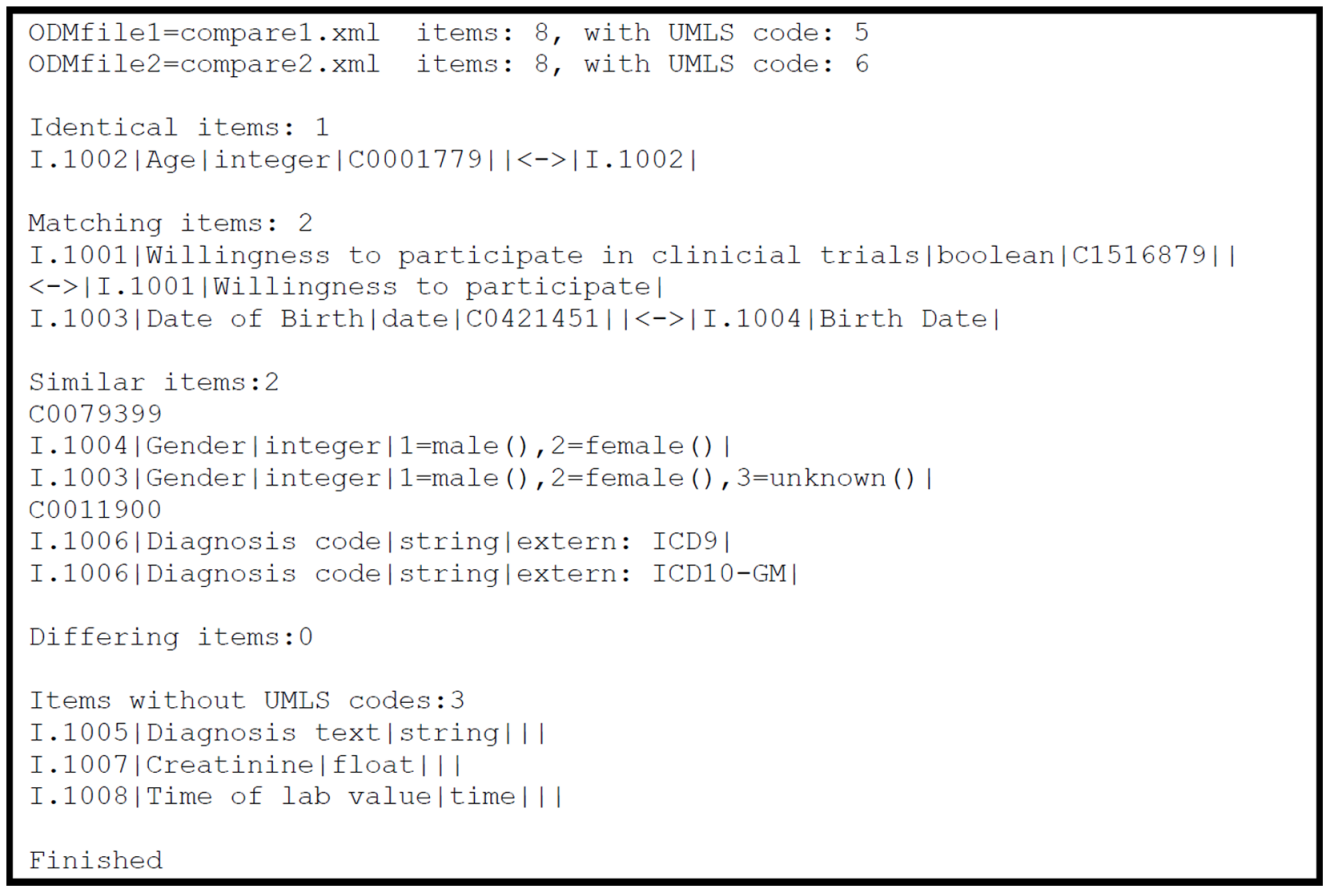
Output of form comparison tool (compareODM). Forms represented by compare1.xml and compare2.xml have 1 identical item, 2 matching items and 2 similar items. 3 items in ODMfile1 do not have an UMLS code assigned; therefore an automatic comparison is not possible. Items are presented with name, value domain and concept code.

Figure 3 shows the output of the reference implementation to compare medical forms for the example provided in figure 1 and 2. Only items with UMLS codes can be compared automatically with this method. To identify items within each form, item object identifier (OID) from each ODM file are provided. The output of this reference implementation can be used by human experts to review similarities and differences between forms either different versions of the same form or two forms from different systems. In addition, this tool can be used to identify “compatible” data items (i.e. identical or matching items) in different forms from where data can be pooled. Similar data items need transformations regarding the value domain before structured data exchange or joint data analysis can be applied.

To further validate our proposed method, a human expert coded two surgical forms (21 items and 31 items) and identified manually 4 identical, 5 matching, 3 similar and 9 differing items. This manual analysis generated the same output like compareODM.

### Evaluation of a Form Set

For the evaluation 7 real medical forms were manually selected, exported in ODM format and stored in a common local folder. The compare-method was applied and results were stored in three matrices.

The forms are related to prostate cancer and cover different areas:

- Two forms are taken from the quality management
- One form contains items for an epidemiological cancer registry
- Four forms are used in the routine care and are taken from a hospital information system

These 7 forms have 285 items in total with an average number of 41 items per form (minimum 5; maximum 87). All items and also the value domains are coded with UMLS codes. The following figures show the visualizations of the result matrices. In total there are 9 images as all 3 result matrices are presented as grid images with absolute numbers, grid image with relative numbers and dendrograms. For reasons of clarity only one of these images is presented per category. All nine images can be found in File S1.

#### Grid Image

**Figure 4:**
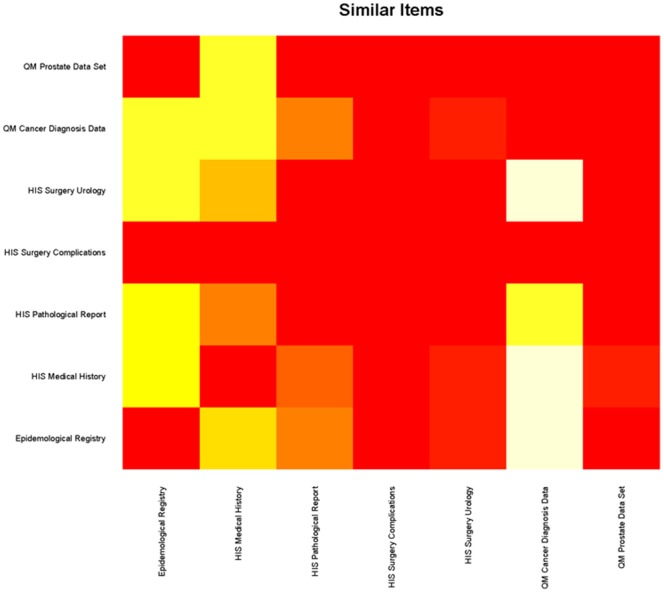
Grid-Image example based on the matrix with comparison results of similar items. This figure shows the absolute number of similar items in a pairwise comparison. Yellow cells represent high number of similar items; red cells represent zero similar items between the two respective forms.

**Figure 5:**
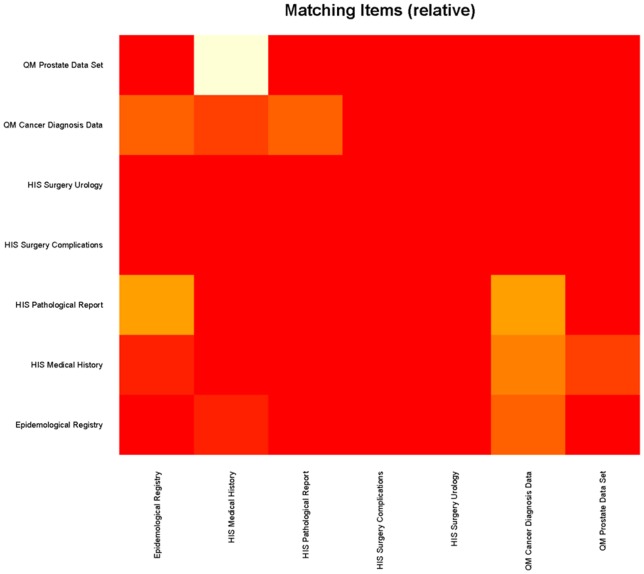
Grid-Image example based on the matrix with comparison results of matching items. This figure shows the relative number of matching items in a pairwise comparison. Yellow cells represent high percentages of similar items; red cells represent low percentages of matching items between the two respective forms.

Figure 4 shows a grid image based on the identical result matrix, figure 5 a grid image regarding matching items. Forms are listed on the x- and y-axes and the similarity between two forms is coded as color information where yellow cells represent high number of identical items, red cells represent zero identical items between the two respective forms.

In addition to the grid image based on absolute numbers a relative grid-image was created as the number of items per form may differ too much and common items in a small form may be underrepresented.

The grid image gives also a good overview on the whole form set which is compared. If all the cells are represented in the same color the forms have many (yellow) or only a few items (red) in common. For more detailed information on the similarity of two forms dendrograms are used.

#### Dendrogram

A dendrogram is useful for a more detailed analysis of the results of the form comparison because also cluster information is added. In addition, the distance between two forms is visualized by the length of the lines (y-axis). The distance is defined by the number of items which the forms have in common.

**Figure 6:**
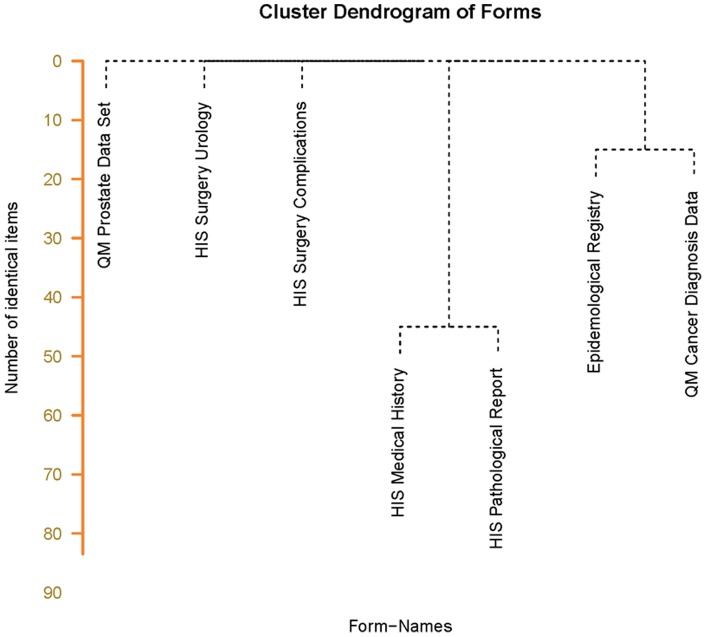
Dendrogram based on the matrix with results of comparison of identical items. This figure shows that similarity between medical history and the pathological report (48 items in common) is higher than between the epidemiological registry form and the quality management form (18 items in common). The other forms do not share identical items.

Figure 6 visualizes the similarity of the form set. There are 48 common items between the medical history form and the pathological report. The form for the *Epidemiological Cancer Registry* shares 16 items with the cancer form from the *Quality Management* while the other three forms have no items in common.

While looking into form details, the *Medical History Form* is a basic cancer form which contains many common items which are could also be found in the *Pathological Report*. The epidemiological cancer registry asks partly for the same items as the quality management. In both cases identical items are at least documented twice from which one documentation step could be avoided by reusing existing documentation. This was visualized by an automated comparison of forms.

## Discussion

As outlined in the introduction there is a huge heterogeneity of medical forms: Thousands of data items in hundreds of different systems. This phenomenon is caused by the complexity of the clinical phenotype (which is inevitable), but also by many independent systems which currently do not allow exchanging metadata of forms. To enable structured data exchange – between different healthcare providers as well as between clinical and research context – more harmonization of forms is urgently needed.

One important step in such a harmonization process of data structures is a systematical comparison of medical forms, including semantic aspects. Simple comparison of item names within forms is not appropriate for several reasons: item names can be defined in different languages (for example English or German) and the meaning of item names depends on the context (for example: “length” can be related to an arm or a leg). For this reason a semantic annotation of items is necessary to support comparison of individual items and forms built from these items.

We propose to use UMLS concept codes as semantic annotations for medical forms, because UMLS is a metathesaurus of almost all medically relevant terminologies. ODM representation of medical forms are selected because ODM is a highly generic, open and vendor neutral standard which is already supported by some EDC systems. In addition, a large set of real medical forms in ODM format is available via a public portal. The semantic interoperability between ODM and HL7 CDA has been shown previously [23]; therefore our approach could be extended to forms in CDA format.

In order to compare a set of forms the three result matrices may be summed up to get a combined indicator for the similarity of the whole set. This could be useful to identify the redundant parts of the documentation as redundancy is not limited to identical items. Especially the dendrogram is helpful to identify a potential of data reuse as it clusters similar forms with a high number of commons items. Two clustered forms in the lower part of the dendrogram share as many items as are indicated by the y-axis. These items are used in each of the clustered forms and indicate which information could be reused by the form or which items could be removed as redundant information.

An open-source reference implementation of *compareODM* is available on the Internet. This manuscript provides details about the proposed form comparison method to collect feedback from the scientific community about feasibility, advantages and disadvantages in other settings at a later point in time. The forms of the repository are also freely available. On a standard PC one single comparison is performed in less than one second. A pairwise comparison of n forms results in nˆ2 comparisons.

### Related work

A literature survey was performed to identify related work. We searched PubMed and Web of Science (Thomson Reuters) using the following keywords: ontological analysis, mapping, semantic annotation, comparison, equivalence, data model, information model, documentation form, document clustering, clinical model, clinical element model, SNOMED CT, archetype, UMLS. The resulting output was reviewed manually. In addition, two experts regarding medical ontologies were contacted to identify similar approaches.

There are several methods to automatically assign semantic codes with relatively high success rates to archetype terms, in particular from SNOMED CT [24], [25], [26]. Other studies focus on ontology editors [27] or ontology development in general [28] rather than comparing documentation forms. There are some publications regarding document clustering [29], [30], but these focus on clustering data, not metadata (data elements). In this literature survey we did not find approaches to automatically compare data models from documentation forms. Given the large number of data elements in medical forms, manual comparison of a larger set of forms is extremely work-intensive and can be done much more efficiently with an automated method like compareODM.

### Limitations

The proposed form comparison method has several limitations:

First, it requires forms in ODM format with UMLS annotations. Forms in ODM format are quite common in EDC systems of clinical trials, but semantic annotations are rarely available. Recently, a portal for semantically annotated medical forms was established [31], which contains several hundred forms in ODM format with UMLS annotations. However, much more tools to build ODM forms with semantic annotations are needed.

Second, automatic comparison of forms based on semantic codes depends on reliable coding of data items, i.e. different human experts should assign the same codes to the same items. UMLS contains approximately 2.7 Mio. concepts (as of June 2012). It has been shown in various settings that intercoder agreement is not perfect, in particular for complex terminologies [32], [33], [34], [35].

## Conclusions

Semantically annotated medical forms in ODM format can be compared automatically to determine identical, matching, similar and differing items. The comparison concept is scalable for sets of real medical forms. Dendrograms provide a good method for the visualization of compared form sets as they cluster forms with common items so that a potential for reusing of information can be identified.

## Supporting Information

File S1 **This file provides additional output of compareODM derived from the 7 medical forms used in the section “Evaluation of a Form Set”.**
(PDF)

## References

[1] Dugas M, Thun S, Frankewitsch T, Heitmann KU (2009) LOINC® Codes for Hospital Information System documents – a case study. J Am Med Inform Assoc. 16(3): 400–3. 10.1197/jamia.M2882 PMC2732230 19261942

[2] Baorto D, Li L, Cimino JJ (2009) Practical experience with the maintenance and auditing of a large medical ontology. J Biomed Inform 42(3): 494–503. 19285569 10.1016/j.jbi.2009.03.005 PMC3508433

[3] Medical Entities Dictionary (MED). http://med.dmi.columbia.edu/ Accessed 2012 Jun 8.

[4] National Cancer Institute (NCI) caBIGTM Form Builder. http://gforge.nci.nih.gov/projects/formbuilder/ Accessed 2012 Jun 8.

[5] EuroRec. http://www.eurorec.org/ Accessed 2012 Jun 10.

[6] ClinicalTrials.gov. http://www.clinicaltrials.gov Accessed 2012 Jun 10.

[7] Weng C, Appelbaum P, Hripcsak G, Kronish I, Busacca L, et al (2012) Using EHRs to integrate research with patient care: promises and challenges. J Am Med Inform Assoc 19(5): 684–687. 22542813 10.1136/amiajnl-2012-000878 PMC3422845

[8] CDISC Operational Data Model (ODM). http://www.cdisc.org/odm/ Accessed 2012 Jun 8.

[9] HL7 CDA. http://www.hl7.org/implement/standards/product_brief.cfm? product_id = 7 Accessed 2012 Jun 12.

[10] Metadata Standards – ISO 11179. http://metadata-standards.org/11179/ Accessed 2014 Feb 4.

[11] Unified Medical Language System (UMLS). http://www.nlm.nih.gov/research/umls/ Accessed 2012 Jun 12.

[12] International Health Terminology Standard Development Organisation (IHTSDO). SNOMED CT. http://www.ihtsdo.org/snomed-ct/ Accessed 2013 Jan 13.

[13] DIMDI. http://www.dimdi.de/ Accessed 2012 Jun 10.

[14] NCI Metathesaurus. http://ncim.nci.nih.gov/ Accessed 2012 Jun 10.

[15] Pathak J, Wang J, Kashyap S, Basford M, Li R, et al (2011) Mapping clinical phenotype data elements to standardized metadata repositories and controlled terminologies: the eMERGE Network experience. J Am Med Inform Assoc 18(4): 376–86. 21597104 10.1136/amiajnl-2010-000061 PMC3128396

[16] Dolin RH, Alschuler L, Boyer S, Beebe C, Behlen FM, et al (2006) HL7 Clinical Document Architecture, Release 2. J Am Med Inform Assoc 13(1): 30–9. 16221939 10.1197/jamia.M1888 PMC1380194

[17] Continuity of Care Document (CCD). http://www.astm.org/Standards/E2369.htm Accessed 2012 Jun 10.

[18] Kush RD. Interoperability Review: EHRs for Clinical Research. http://www.amia.org/news-and-publications/volume-2-number-2/interoperability-review-2 Accessed 2012 Jun 12.

[19] Dziuballe P, Breil B, Fritz F, Dugas M (2012) Interoperability in Clinical Research: From Metadata Registries to Semantically Annotated CDISC ODM. Stud Health Technol Inform 180: 564–8. 22874254

[20] R Statistical Programming Language. http://www.r-project.org/ Accessed 2012 Jun 10.

[21] Breil B, Kenneweg J, Fritz F, Bruland P, Doods J, et al (2012) Multilingual Medical Data Models in ODM Format. A Novel Form-based Approach to Semantic Interoperability between Routine Healthcare and Clinical Research. ACI 3(3): 276–289. 23620720 10.4338/ACI-2012-03-RA-0011 PMC3613023

[22] Hastie T, Tibshirani R, Friedman J. The elements of statistical learning, http://www-stat.stanford.edu/~tibs/ElemStatLearn/ p.521 ff.

[23] El Fadly A, Daniel C, Bousquet C, Dart T, Lastic PY, et al (2007) Electronic Healthcare Record and clinical research in cardiovascular radiology. HL7 CDA and CDISC ODM interoperability. AMIA Annu Symp Proc 11: 216–20. PMC2655824 18693829

[24] Meizoso García M, Iglesias Allones JL, Martínez Hernández D, Taboada Iglesias MJ (2012) Semantic similarity-based alignment between clinical archetypes and SNOMED CT: an application to observations. Int J Med Inform 81(8): 566–78. 22421520 10.1016/j.ijmedinf.2012.02.007

[25] Yu S, Berry D, Bisbal J (2012) Clinical coverage of an archetype repository over SNOMED-CT. J Biomed Inform 45(3): 408–18. 22200680 10.1016/j.jbi.2011.12.001

[26] Allones JL, Penas D, Taboada M, Martinez D, Tellado S (2013) A study of semantic proximity between archetype terms based on SNOMED CT relationships. Lecture Notes in Computer Science 7738:98–112. In: Process Support and Knowledge Representation in Health Care. BPM 2012 Joint Workshop, ProHealth 2012/KR4HC 2012, Tallinn, Estonia (editors: Lenz R, Miksch S, Peleg M, Reichert M, Riaño D, ten Teije A).

[27] Schober D, Tudose I, Svatek V, Boeker MJ (2012) OntoCheck: verifying ontology naming conventions and metadata completeness in Protégé 4. Biomed Semantics 21 3 Suppl 2S4. 10.1186/2041-1480-3-S2-S4 PMC3448530 23046606

[28] Schulz S, Beisswanger E, van den Hoek L, Bodenreider O, van Mulligen EM (2009) Alignment of the UMLS semantic network with BioTop: methodology and assessment. Bioinformatics 25(12): i69–76. 19478019 10.1093/bioinformatics/btp194 PMC2687948

[29] Fung BCM, Wang K, Ester M (2003) Hierarchical Document Clustering Using Frequent Itemsets. In: Proc. Siam international conference on data mining 2003 (editors: Barbara D, Kamath C), pages 59–70.

[30] Steinbach M, Karypis G, Kumar V. A comparison of document clustering techniques. KDD Workshop on Text Mining 2000. http://glaros.dtc.umn.edu/gkhome/fetch/papers/docclusterKDDTMW00.pdf? Accessed 2013 May 7.

[31] Medical Data Models. http://www.medical-data-models.org/ Accessed 2012 Jun 10.

[32] Hwang JC, Yu AC, Casper DS, Starren J, Cimino JJ, et al (2006) Representation of ophthalmology concepts by electronic systems: intercoder agreement among physicians using controlled terminologies. Ophthalmology 113(4): 511–9. 16488013 10.1016/j.ophtha.2006.01.017

[33] Olsen NS, Shorrock ST (2010) Evaluation of the HFACS-ADF safety classification system: inter-coder consensus and intra-coder consistency. Accid Anal Prev 42(2): 437–44. 20159064 10.1016/j.aap.2009.09.005

[34] Chiang MF, Hwang JC, Yu AC, Casper DS, Cimino JJ, et al.. (2006) Reliability of SNOMED-CT coding by three physicians using two terminology browsers. AMIA Annu Symp Proc 131–5. PMC1839418 17238317

[35] Andrews JE, Patrick TB, Richesson RL, Brown H, Krischer JP (2008) Comparing heterogeneous SNOMED CT coding of clinical research concepts by examining normalized expressions. Biomedical Informatics 41: 1062–1069. 18328789 10.1016/j.jbi.2008.01.010 PMC2605270

