# Supplementary material for: Automated UMLS-Based Comparison of Medical Forms
Source: PLoS One. 2013 Jul 4;8(7):e67883. doi: 10.1371/journal.pone.0067883 (PMC3701617; doi:10.1371/journal.pone.0067883)
Supplement: File S1 — This file provides additional output of compareODM derived from the 7 medical forms used in the section “Evaluation of a Form Set”. (PDF) [file pone.0067883.s001.pdf]

UMLS−based Comparison of Medical Forms

Results from: 07.02.2013

Number of Forms: 7

Number of Items: 285

Min. Number of Items per form: 5

Max. Number of Items per form: 87

Average Number of Items per form: 41

Domain: Prostate cancer

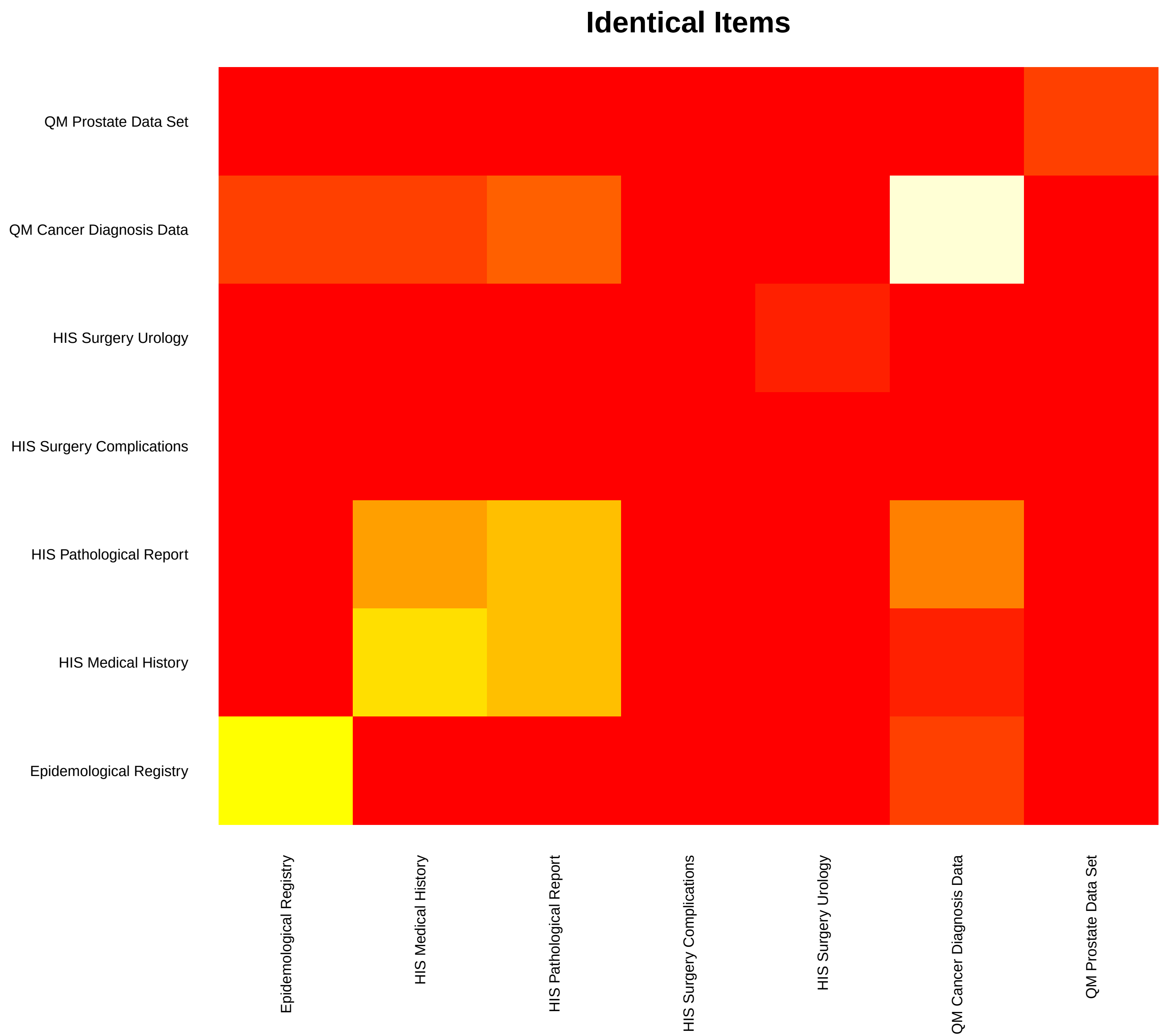
Identical Items
QM Prostate Data Set
QM Cancer Diagnosis Data
HIS Surgery Urology
HIS Surgery Complications
HIS Pathological Report
HIS Medical History
Epidemological Registry
Epidemological Registry
HIS Medical History
HIS Pathological Report
HIS Surgery Complications
HIS Surgery Urology
QM Cancer Diagnosis Data
QM Prostate Data Set

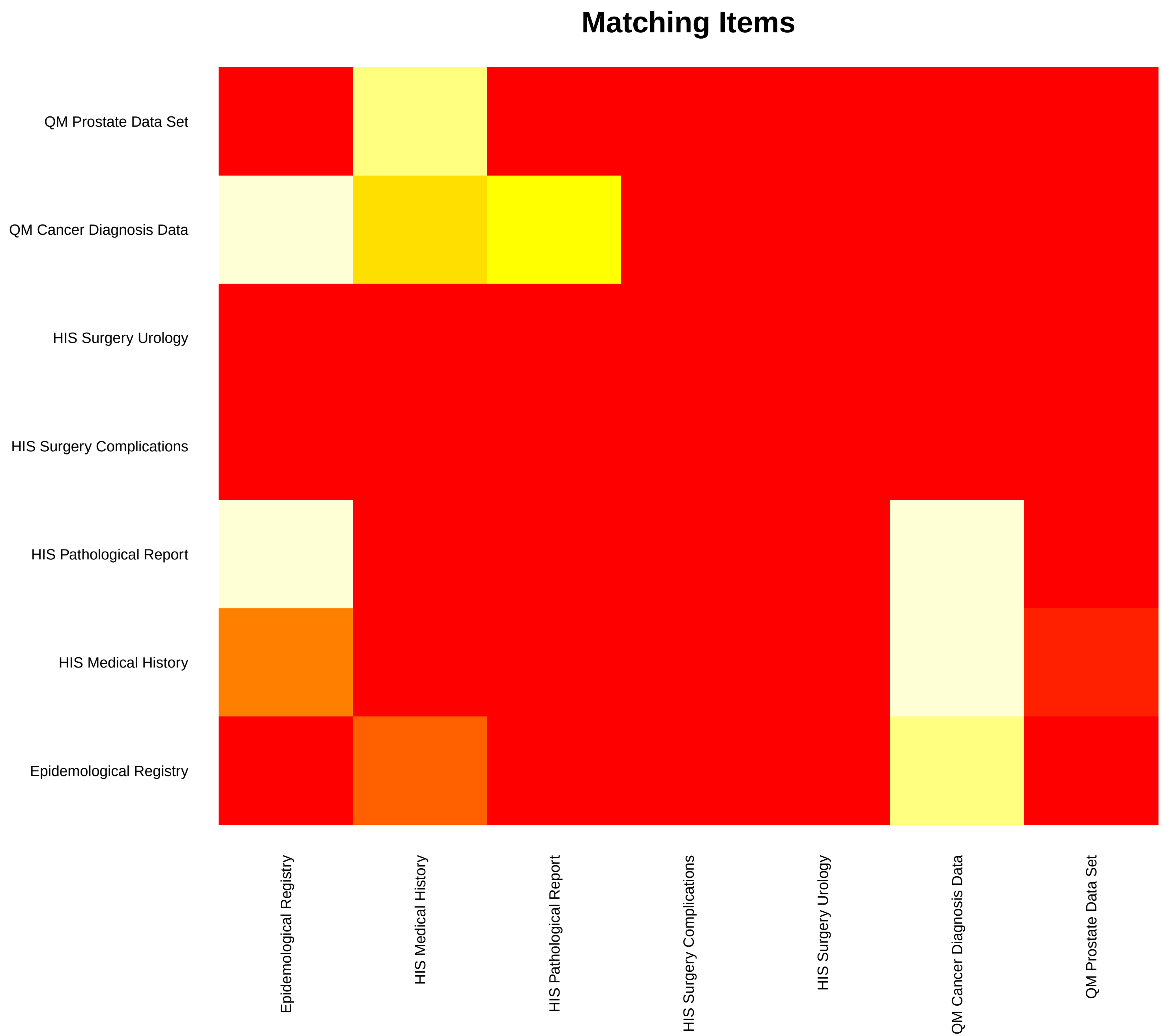
Matching Items
QM Prostate Data Set
QM Cancer Diagnosis Data
HIS Surgery Urology
HIS Surgery Complications
HIS Pathological Report
HIS Medical History
Epidemological Registry
Epidemological Registry
HIS Medical History
HIS Pathological Report
HIS Surgery Complications
HIS Surgery Urology
QM Cancer Diagnosis Data
QM Prostate Data Set

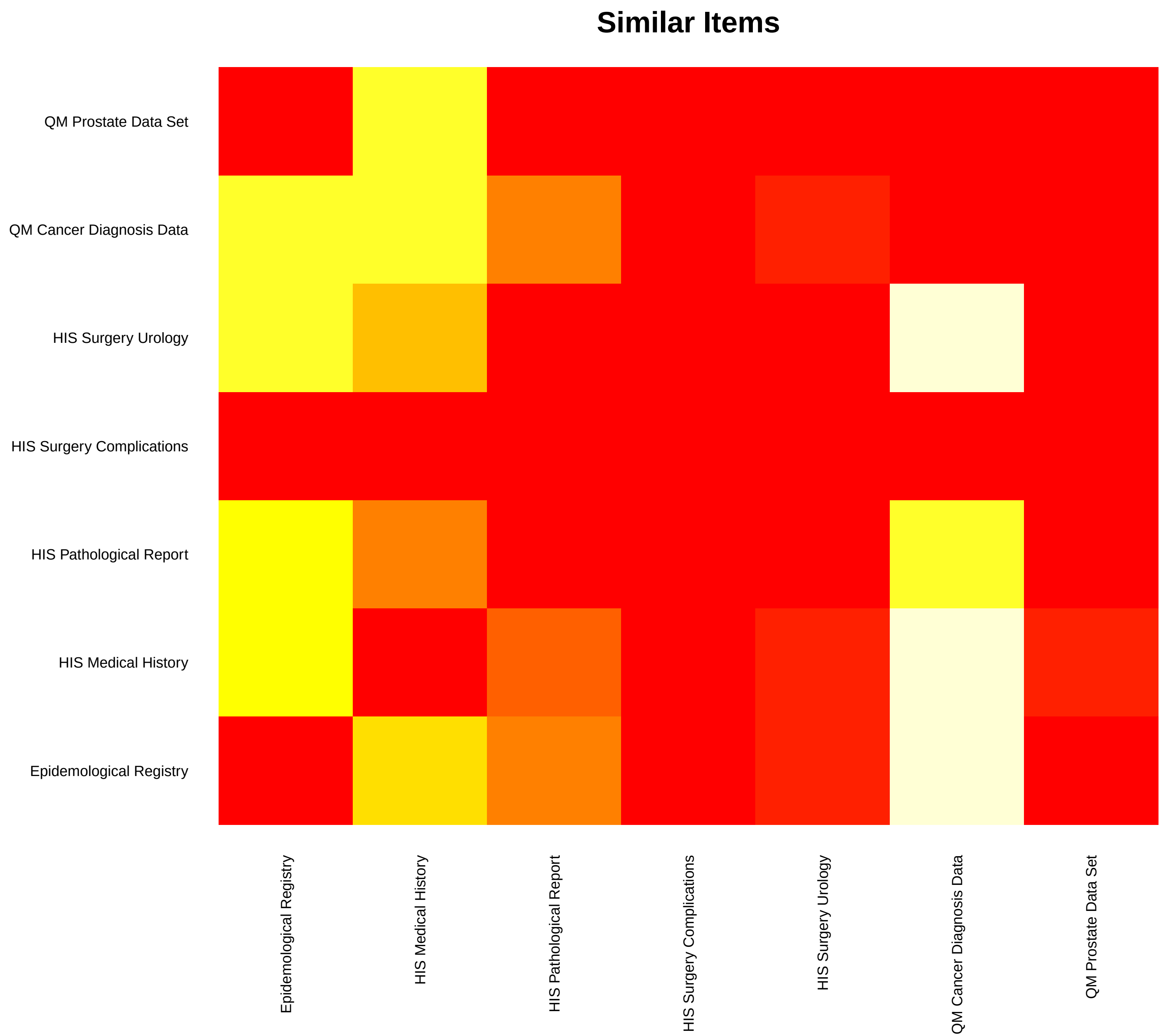
Similar Items
QM Prostate Data Set
QM Cancer Diagnosis Data
HIS Surgery Urology
HIS Surgery Complications
HIS Pathological Report
HIS Medical History
Epidemological Registry
Epidemological Registry
HIS Medical History
HIS Pathological Report
HIS Surgery Complications
HIS Surgery Urology
QM Cancer Diagnosis Data
QM Prostate Data Set

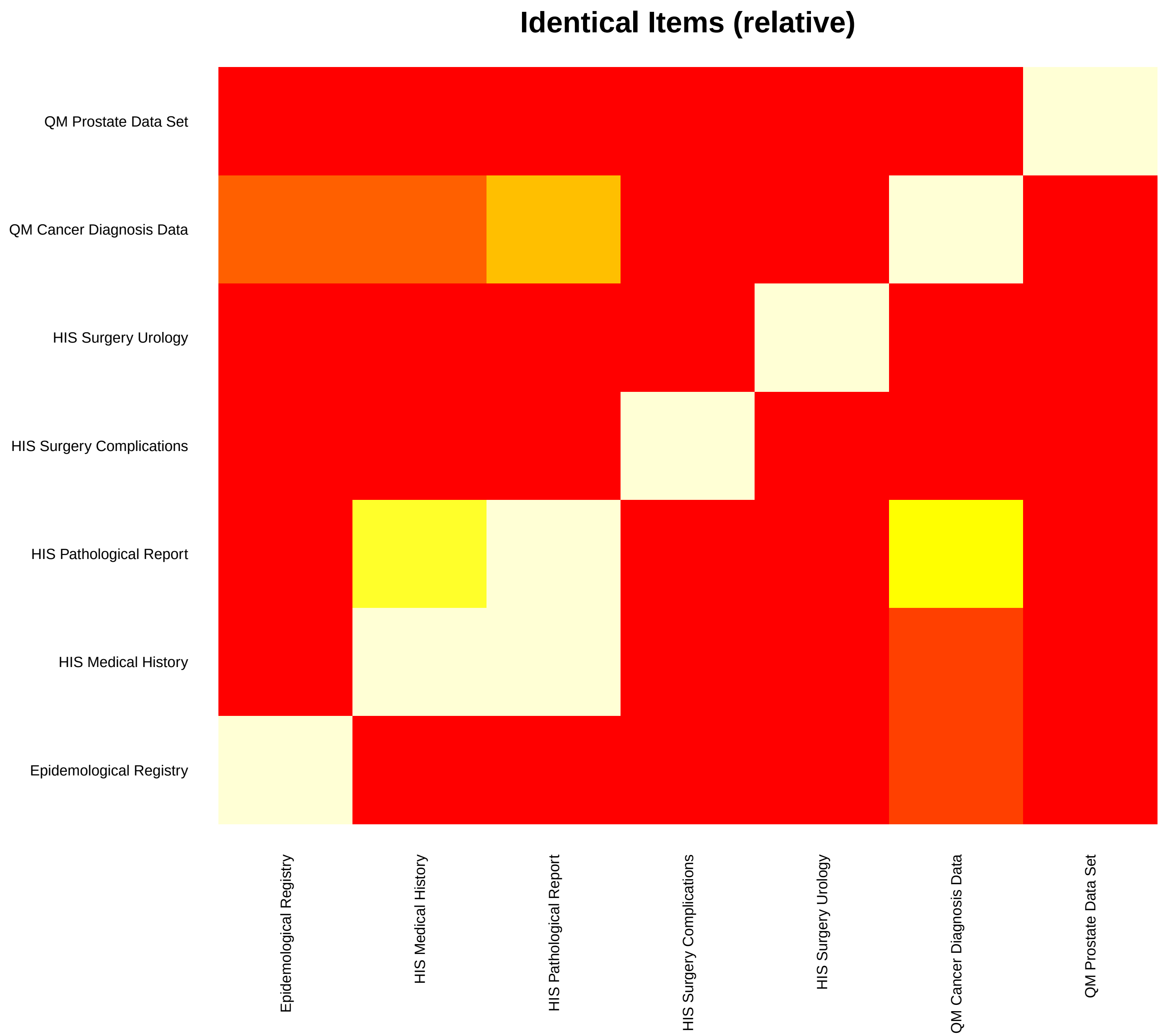
Identical Items (relative)
QM Prostate Data Set
QM Cancer Diagnosis Data
HIS Surgery Urology
HIS Surgery Complications
HIS Pathological Report
HIS Medical History
Epidemological Registry
Epidemological Registry
HIS Medical History
HIS Pathological Report
HIS Surgery Complications
HIS Surgery Urology
QM Cancer Diagnosis Data
QM Prostate Data Set

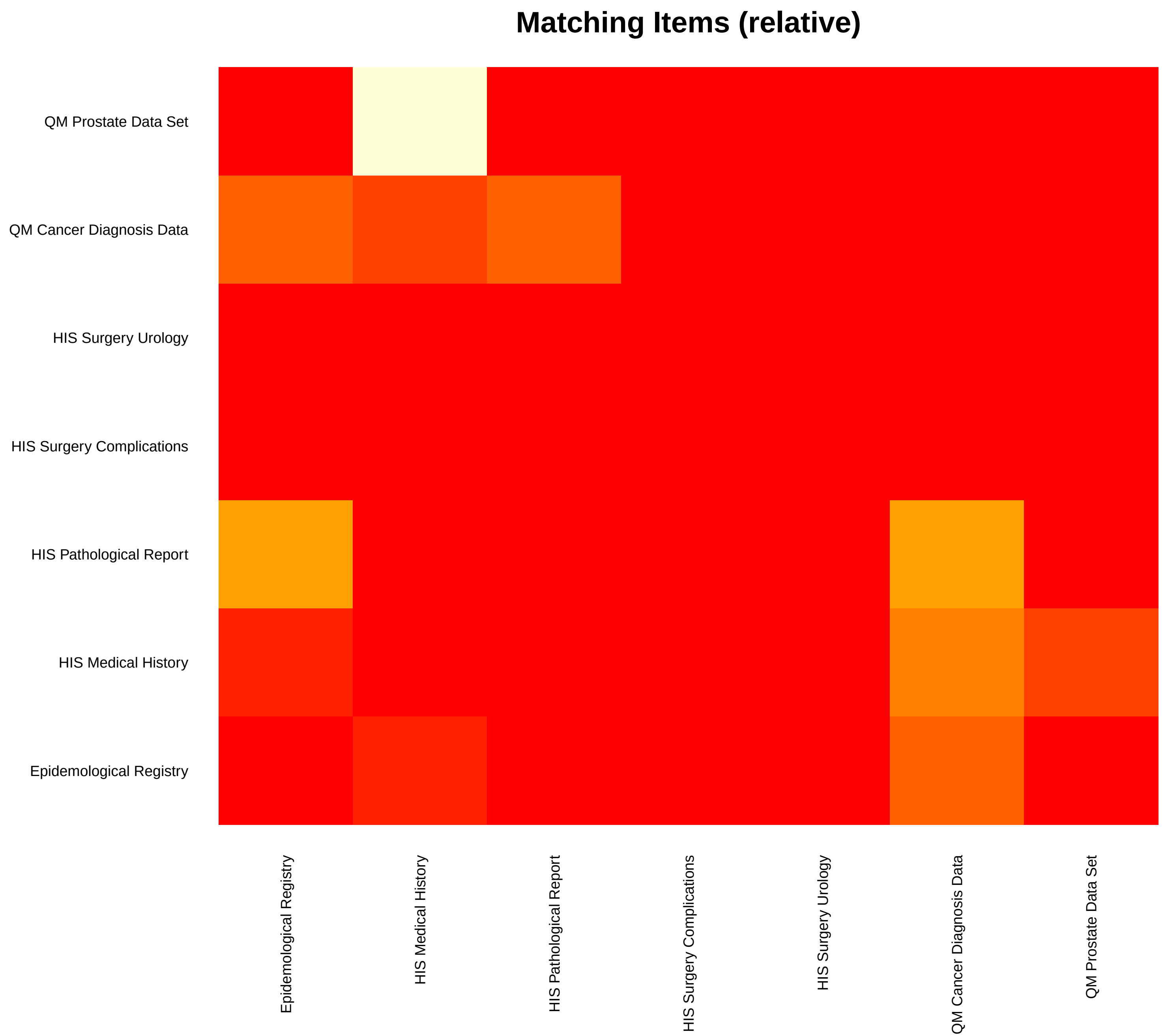
Matching Items (relative)
QM Prostate Data Set
QM Cancer Diagnosis Data
HIS Surgery Urology
HIS Surgery Complications
HIS Pathological Report
HIS Medical History
Epidemological Registry
Epidemological Registry
HIS Medical History
HIS Pathological Report
HIS Surgery Complications
HIS Surgery Urology
QM Cancer Diagnosis Data
QM Prostate Data Set

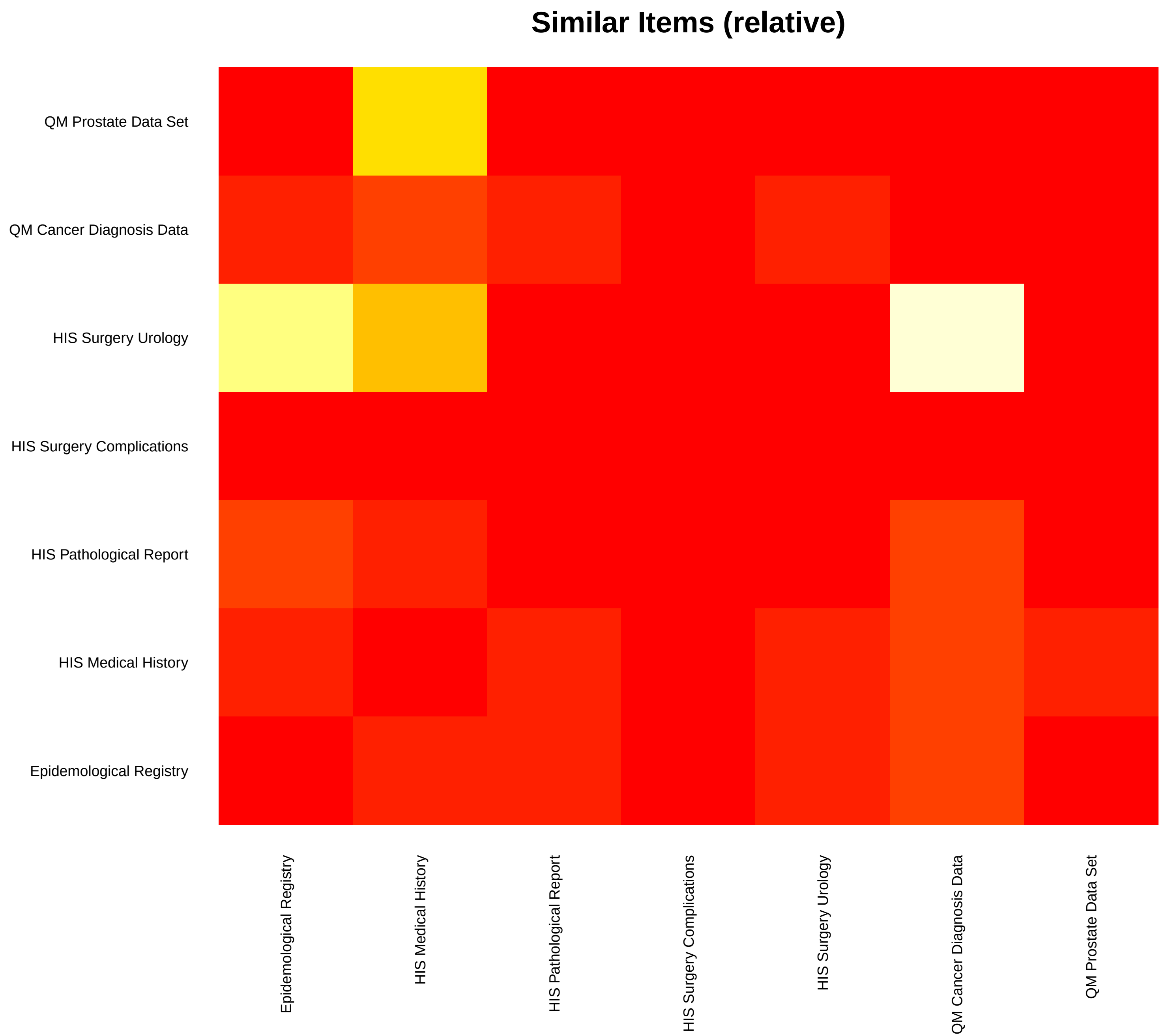
Similar Items (relative)
QM Prostate Data Set
QM Cancer Diagnosis Data
HIS Surgery Urology
HIS Surgery Complications
HIS Pathological Report
HIS Medical History
Epidemological Registry
Epidemological Registry
HIS Medical History
HIS Pathological Report
HIS Surgery Complications
HIS Surgery Urology
QM Cancer Diagnosis Data
QM Prostate Data Set

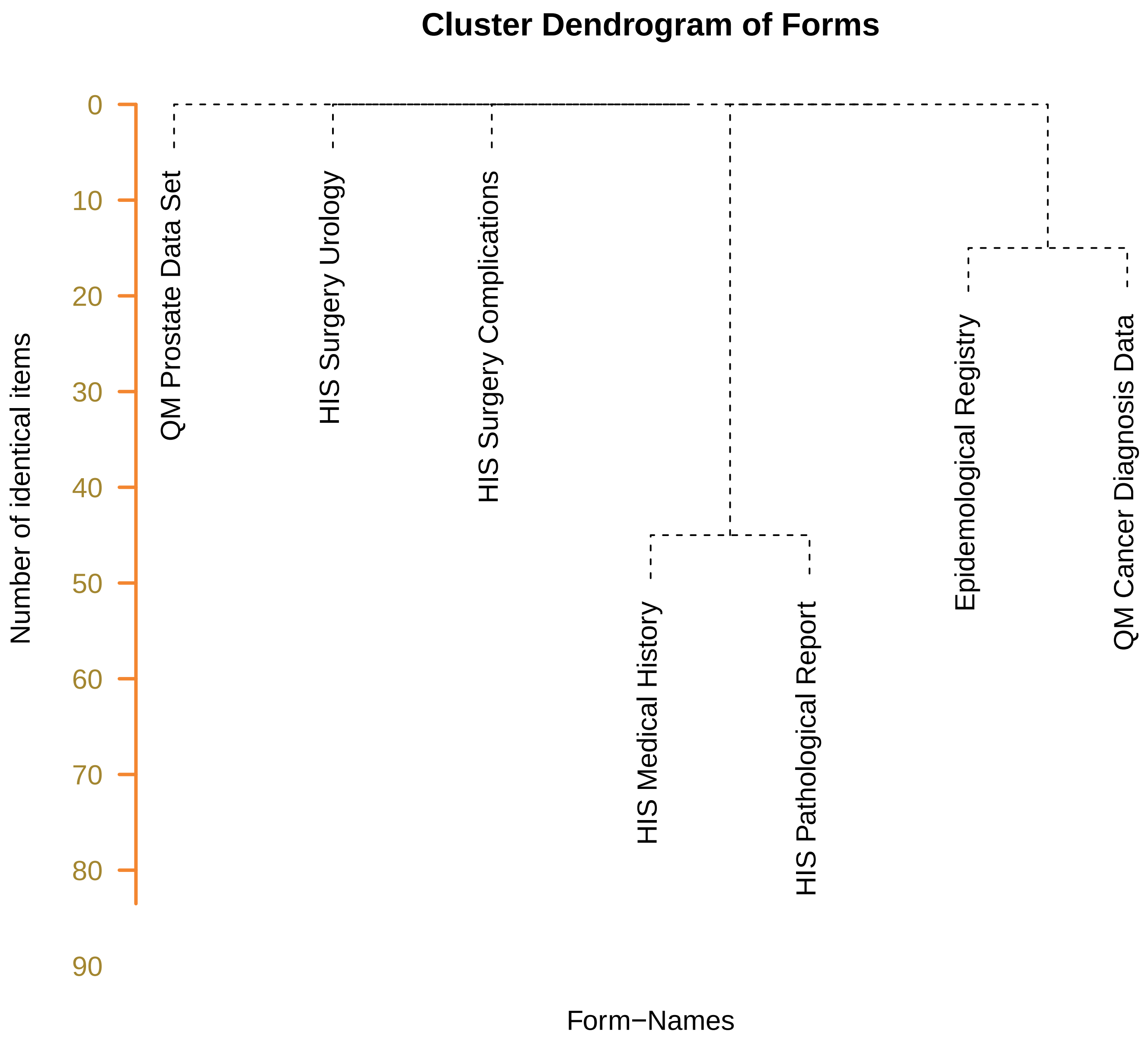
Cluster Dendrogram of Forms
Number of identical items
0
10
20
30
40
50
60
70
80
90
QM Prostate Data Set
HIS Surgery Urology
HIS Surgery Complications
HIS Medical History
HIS Pathological Report
Epidemological Registry
QM Cancer Diagnosis Data
Form−Names

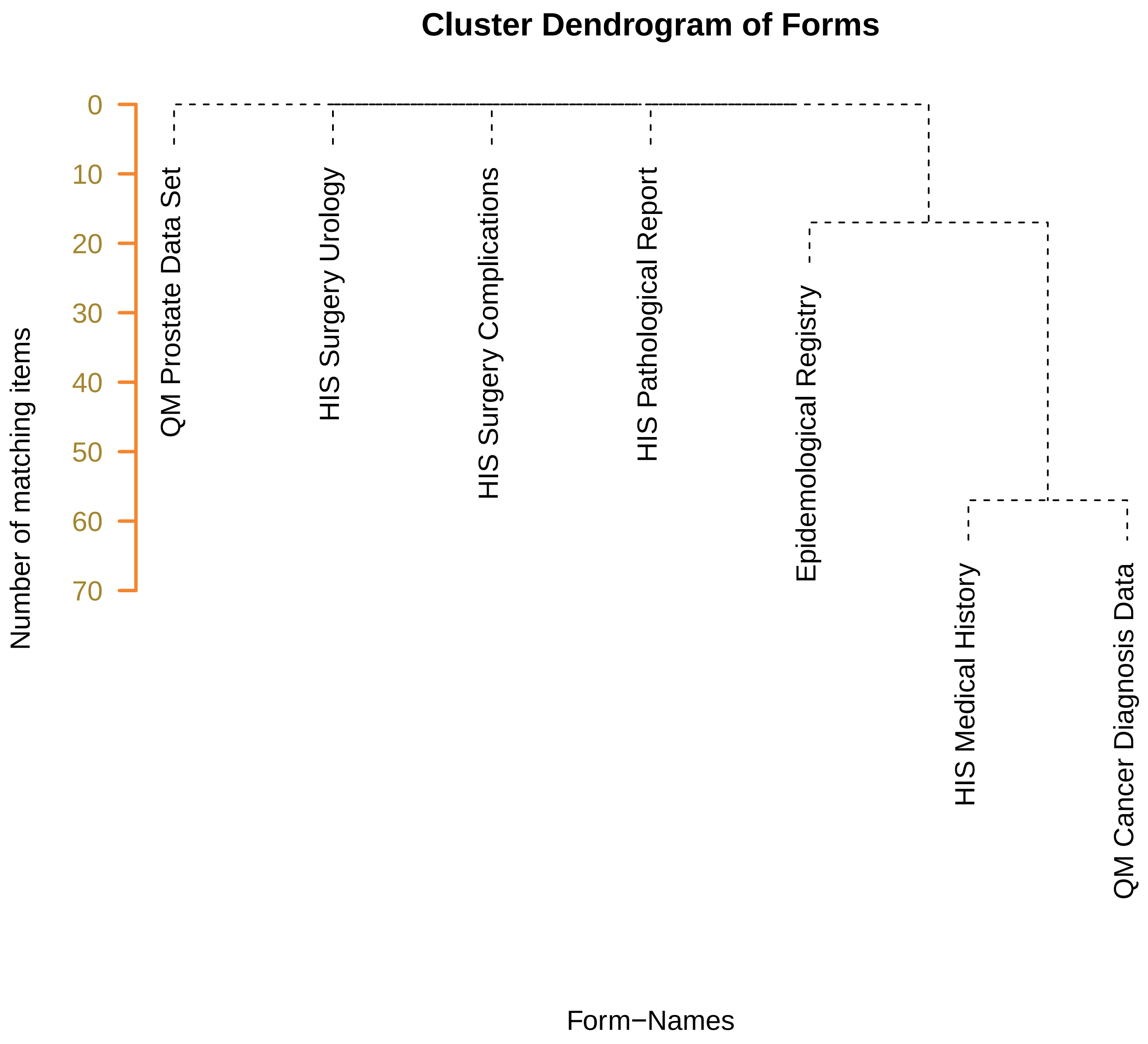

Cluster Dendrogram of Forms
0
10
20
30
40
50
60
70
Number of matching items
QM Prostate Data Set
HIS Surgery Urology
HIS Surgery Complications
HIS Pathological Report
Epidemological Registry
HIS Medical History
QM Cancer Diagnosis Data
Form−Names

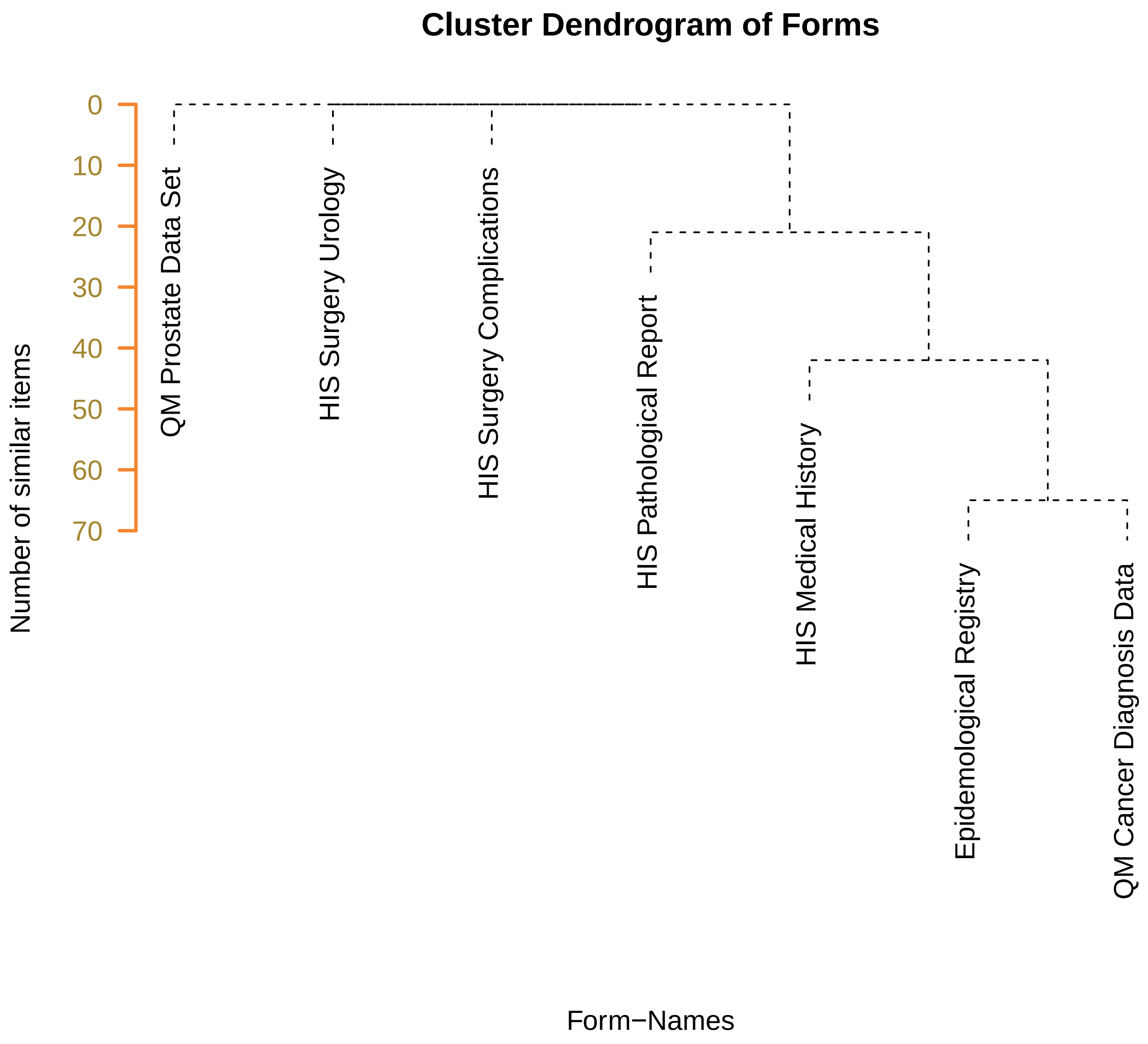
Cluster Dendrogram of Forms
Number of similar items
0
10
20
30
40
50
60
70
QM Prostate Data Set
HIS Surgery Urology
HIS Surgery Complications
HIS Pathological Report
HIS Medical History
Epidemiological Registry
QM Cancer Diagnosis Data
Form−Names
